# Identification of Proteins from Interstitium of Trapezius Muscle in Women with Chronic Myalgia Using Microdialysis in Combination with Proteomics

**DOI:** 10.1371/journal.pone.0052560

**Published:** 2012-12-27

**Authors:** Patrik Olausson, Björn Gerdle, Nazdar Ghafouri, Britt Larsson, Bijar Ghafouri

**Affiliations:** 1 Rehabilitation Medicine, Department of Medicine and Health Sciences, Faculty of Health Sciences, University of Linköping and Pain- and Rehabilitation Centre, County Council, Linköping, Sweden; 2 Department of Pharmacology and Clinical Neuroscience, Umeå University, Umeå, Sweden; 3 Occupational and Environmental Medicine, Department of Clinical and Experimental Medicine, Faculty of Health Sciences, Linköping University and Centre of Occupational and Environmental Medicine, County Council, Linköping, Sweden; University of Sao Paulo, Brazil

## Abstract

**Background:**

Microdialysis (MD) of the trapezius muscle has been an attractive technique to investigating small molecules and metabolites in chronic musculoskeletal pain in human. Large biomolecules such as proteins also cross the dialysis membrane of the catheters. In this study we have applied *in vivo* MD in combination with two dimensional gel electrophoresis (2-DE) and mass spectrometry to identify proteins in the extracellular fluid of the trapezius muscle.

**Materials and Methods:**

Dialysate from women with chronic trapezius myalgia (TM; n = 37), women with chronic wide spread pain (CWP; n = 18) and healthy controls (CON; n = 22) was collected from the trapezius muscle using a catheter with a cut-off point of 100 kDa. Proteins were separated by two-dimensional gel electrophoresis and visualized by silver staining. Detected proteins were identified by nano liquid chromatography in combination with tandem mass spectrometry.

**Results:**

Ninety-seven protein spots were identified from the interstitial fluid of the trapezius muscle; 48 proteins in TM and 30 proteins in CWP had concentrations at least two-fold higher or lower than in CON. The identified proteins pertain to several functional classes, e.g., proteins involved in inflammatory responses. Several of the identified proteins are known to be involved in processes of pain such as: creatine kinase, nerve growth factor, carbonic anhydrase, myoglobin, fatty acid binding protein and actin aortic smooth muscle.

**Conclusions:**

In this study, by using in vivo microdialysis in combination with proteomics a large number of proteins in muscle interstitium have been identified. Several of the identified proteins were at least two-fold higher or lower in chronic pain patients. The applied techniques open up for the possibility of investigating protein changes associated with nociceptive processes of chronic myalgia.

## Introduction

The risk for developing chronic trapezius myalgia (TM) is increased in several occupations with work tasks such as high exposure to awkward working positions, sustained static and repetitive movements of the arms and movements with high precision demands [1], [2]. Various but often ineffective treatment interventions are frequently implemented [3]. For some of the patients the pain will spread and a chronic widespread pain condition (CWP) can develop [4]. The pathophysiological mechanisms behind TM and CWP are poorly understood.

Microdialysis (MD) mimics the function of a capillary blood vessel by perfusing a thin dialysis tube implanted in the tissue with a physiological saline solution [5]. Substances can pass across the dialysis membrane along the concentration gradient. The dialysate is analyzed and reflects the composition of the extra cellular fluid where nociceptor free nerve endings terminate. Metabolic, algesic and potential nociception/pain inhibitory substances have been studied in chronic myalgia using MD [6]. Significant increases e.g., in serotonin, glutamate, lactate, pyruvate, *N*-stearoylethanolamine and palmitoylethanolamide have been reported; for a review see [6]. In the majority of hitherto performed studies one or a few small biochemical substances have been exclusively in focus. There are a few attempts to analyze larger molecules, e.g., proteins, with various results [7]–[10]. Proteins can act as signal substances, activate the formation of algesics and control nociceptive processes. Proteomics, defined as the science and the methodology of investigating the proteome [11] has been suggested as a useful technique in order to identify biomarkers of pain conditions [12]–[14]. Two-dimensional gel electrophoresis (2-DE) is a widely used technique to separate organic substances such as proteins in different tissues [15]. Proteomics have been applied to pain research to identify protein changes in different pain conditions. The proteome has been investigated in animals comparing inflammatory and neuropathic pain [16]. The proteome of the cerebrospinal fluid in healthy subjects, in patients with herniated disc and idiopathic back pain have been studied [17]–[19]. Also, nerve samples from patients with complex regional pain syndrome (CRPS) have been analyzed using proteomics in order to detect up/down regulated proteins [20]. Proteomics of serum have been used for the identification of possible biomarkers of chronic endometriosis [21], [22]. To our knowledge this technique has not been used for investigating potential alterations in muscle interstitium. In this study we have applied *in vivo* MD in combination with 2-DE and mass spectrometry to identify proteins in the interstitial fluid of the trapezius muscle in healthy subjects, in patients with chronic trapezius myalgia and in chronic widespread pain.

## Materials and Methods

### Subjects

37 women with chronic trapezius myalgia (TM), 18 women with chronic wide spread pain (CWP) and 22 healthy women (CON) were included in this study.Women were chosen as subjects in the present study due to the higher prevalence of chronic pain in women than in men according to community based epidemiological studies. Moreover the majority (approx. 65%) of patients referred to our clinical department (Pain- and rehabilitation Centre, University Hospital, Linköping, Sweden) are women. In future studies we will investigate men. The criteria for chronic trapezius myalgia have been described in our earlier studies [23]–[26]. The ACR criteria were used for the diagnosis of CWP [27]. No significant group differences in age (CON: 43.5±10.2 years, TM: 43.6±9.3 years and CWP: 47.9±9.6 years), height (CON: 168±7cm, TM: 166±6 cm and CWP: 167±5 cm) and weight (CON: 67.1±10.3 kg, TM: 67.0±12.2 kg and CWP: 75.8±17.3 kg) existed. The pain intensity was rated immediately before the experiment using a visual analogue scale (VAS) and as expected significant group differences existed (CON: 0±0 mm, TM: 15±16 mm and CWP: 43±22 mm; p<0.001).

Patients were instructed not to drink any beverages with caffeine on the day of the study, not to smoke and to avoid NSAID-medication the week before the study. The participants arrived at the clinic in the morning after having eaten breakfast. A brief interview was then made by one of the physicians checking that the instructions with respect to the different instructions had been followed. All subjects reported that they had followed the instructions. During the study, they were not allowed to eat, but they could drink water.

All participants gave their informed written consent before the start of the study. The study was approved by the Ethical Committee of Linköping University (Dnr: M10-08).

### Microdialysis

The MD technique used has been described in detail in our previous studies [23]–[25], [28], [29]; here is given a summary. The skin and the subcutaneous tissues above, where the catheter entered were anaesthetized with a local injection (0.5 ml) of Xylocaine (20 mg/ml) without adrenaline, and care was taken not to anaesthetize the underlying muscle. As guidance the insertion of the microdialysis catheter, which was inserted in the middle third of the upper part of the trapezius muscle in the direction lateral to medial were preceded by ultrasound investigation of distance between the skin and the trapezius muscle and the width of the muscle. Using the SENIAM landmarks [30] the midpoint of the line between the spine of 7th cervical vertebra and the acromion was defined as the midpoint of the descending trapezius. The commercially available microdialysis catheter (CMA 71, cut-off points of 100 kDa, CMA Microdialysis AB, Solna, Sweden; membrane 30 mm length, 0.5 mm diameter) were inserted into the pars descendens of the trapezius muscle at half the distance between the processus spinosus of seventh cervical spine and the lateral end of the acromion. Typically, a brief involuntary contraction and change of resistance were perceived when the tip of the insertion needle of the catheter entered the fascia and the muscle. The catheters were placed in the trapezius muscle parallel to the muscle fibers and perfused with a high-precision syringe pump (CMA 107; CMA/Microdialysis AB, Stockholm, Sweden) at a rate of 5 µl/min with a Ringer acetate solution (Fresenius Kabi AB, Uppsala, Sweden) containing 3 mM glucose and 0.5 mM lactate in order to mimic the interstitial environment of the muscle [31].

Immediately after the insertion of catheters participants rested comfortably in an armchair for a 120 minutes trauma period to allow the tissue to recover from possible changes in the interstitial environment induced. Samples from trauma period were discarded and after the trauma period, sampling was performed at every 20 minutes in a period of 100 minutes. In the present study dialysate from the time period 140–200 min was used. The samples were stored on ice to prevent protease activation. The samples were then stored as aliquots in −70°C until analysis. All vials were weighted before the experiment started and after each 20 minutes interval in order to confirm that sampling and fluid recovery (FR) was working according to the perfusion rate set. Vials with visible sign of hemolysis were discarded.

### Two dimensional gel electrophoresis (2-DE)

Dialysate samples from the time period of 140–200 minutes were used for 2-DE analysis. Between 10–20 µl of dialysate from each subjects in TM, CWP and CON group were pooled. Samples were desalted by gel filtration (PD-10 column, GE Healthcare) into 12 mM ammonium bicarbonate, pH 7.1. Proteins were lyophilized and dissolved in 0.20 ml urea sample solution according to Görg [15]. 2-DE was performed in a horizontal 2-DE setup (IPGphore and Multiphore from GE Healthcare), as described in detail previously [32] and essentially according to Görg [15]. The samples (containing 50 µg protein for analytical gels and 300 µg for preparative gel) were applied by in-gel rehydration (according to the manufacturer's instructions) for 12 h using low voltage (30 V) in pH 3–10 L IPGs. The proteins were then focused for up to 32 000 Vhs at a maximum voltage of 8000 V. IPGs were either used immediately for second dimensional analysis, or stored at −70°C until analyzed. The second dimension (SDS-PAGE) was carried out by transferring the proteins to gradient gels cast on GelBond PAG film (0.5/180/245 mm, 11–18%T, 1.5%C, 33–0% glycerol) running at 30 mA and up to 1000 V for about 5 h.

### Staining and image analysis

In analytical gels separated proteins were detected by silver staining with a detection limit of about 5 ng/spot [33]. Proteins picked for MS analysis were mostly fluorescently stained with SYPRO Ruby. Fluorescent staining was performed according to the manufacturer's staining protocol (SYPRO Ruby protein gel stain web site: www.probes.com). After SDS-PAGE gels were fixed using 10% methanol/7% acetic acid solution for 30 min and then incubated in 400 ml SYPRO Ruby protein gel stain solution overnight. Gels were washed and placed in deionized water. All staining and washing steps were performed with continuous gentle agitation. The protein patterns of silver stained analytical gels were analyzed as digitized images, using a CCD camera in combination with a computerized imaging 12-bit system designed for evaluation of 2-DE patterns. The amount of protein in a spot was assessed as background corrected optical density, integrated over all pixels in the spot and expressed as integrated optical density (IOD). In order to correct for differences in total silver stain intensity between different 2-DE images, the amounts of the compared protein spots were quantified as optical density for individual spot per total protein intensity of all spots in the same gel. Thereby ppm-values (parts per million) for all proteins were generated that were evaluated for differences between the groups.

### In-gel digestion by trypsin

In the gel used for protein identification 300 µg proteins were loaded and analyzed as above. Protein spots were excised using a homemade spot picker. The picked protein spots were digested with trypsin (Promega/SDS Biosciences, Falkenberg, Sweden). Briefly, the gel pieces were washed with a mixture of acetonitrile/ammonium bicarbonate, dehydrated with acetonitrile and incubated with 30 µl of 20 µg/ml trypsin overnight at 37°C. Silver stained protein spots were destained with 15 mM potassium ferricyanide/50 mM sodium thiosulfate as described previously [34] before trypsination. The supernatant was transferred to a new tube and the peptides further extracted from the gel by incubation in 50% acetonitrile/5% trifluoroacetic acid for about 3 hours at room temperature during constant mixing. The supernatant obtained by the two steps pooled, dried by SpeedVac.

### Protein identification by LC-MS/MS

The dried tryptic samples from fluorescently stained proteins were dissolved in 6 µl of 0.1% formic acid. Peptides were analyzed using an on-line nano-flow HPLC system (EASY-nLC; Proxeon, Bruker Daltonics) in conjugation with the mass spectrometer HCTultra PTM Discovery System (Bruker Daltonics). A 100 mm×75 µm C18 column was used for separation at a flow rate 300 nL/min. The gradient buffers used were 0.1% formic acid in water (buffer A) and 0.1% formic acid in acetonitrile (buffer B) and a linear gradient from 0–100% buffer B in 40 min was used for separation. The automated online tandem MS analysis was performed using collision induced dissociation of peptide ions.

### Protein identification by MALDI-TOF

The dried tryptic samples from silver stained proteins were dissolved in 4 µl of 0.1% trifluroacetic acid (TFA). The peptides were mixed 1∶1 with matrix solutions consisting of dihydroxybenzoic acid (DHB) (0.04 g/ml) in 70% ACN/0.3% TFA, and 1 µl was then spotted on the target plate (stainless-steel plate). Analysis of peptide masses was performed as described previously [25] using MALDI–TOF MS (Voyager-DE PRO, Applied Biosystems, Foster City, CA, USA).

### Database searches

LC-MS/MS spectra were processed by Bruker Daltonics DataAnalysis 3.4 (Bruker Daltonics, Bremen, Germany) and resulting MS/MS data were searched in NCBInr and Swiss-Prot database on MASCOT server (www.matrixscience.com). Database search parameters were set as follows: the enzyme trypsin was used; up to one missed cleavage was allowed; fixed modification included were carbamidomethylation of cysteine and oxidation of methionine; mass tolerance for MS precursor ion was 0.8 Da and for MS/MS fragment ion was 0.6 Da; and charge states were varied. Criteria for identification of a protein were at least 3 peptides of the protein should be identified with a MASCOT score over 25 and an expectation value <1.

The mass list generated from the major peaks of the MALDI spectra was submitted to a database search (NCBI or SWISS-PROT) using MS-FIT search engines. Restrictions were placed on species (Human), mass tolerance (50 ppm), maximum missed cleavages by trypsin (up to 1) and cysteine modification by carbamidomethylation.

### Statistics

For comparison of group differences concerning anthropometric data and pain intensity the Kruskal Wallis test was applied using IBM SPSS (version 20.0); p<0.05 was considered significant.

## Results

### Protein concentration

The total protein concentrations were measured before 2-DE analysis; 74 µg/ml in the TM pool, 55 µg/ml in the CWP pool and 59 µg/ml in the CON pool. The samples were desalted, concentrated and 50 µg protein from each group could be analyzed by 2-DE.

### 2-DE analysis

**Figure 1:**
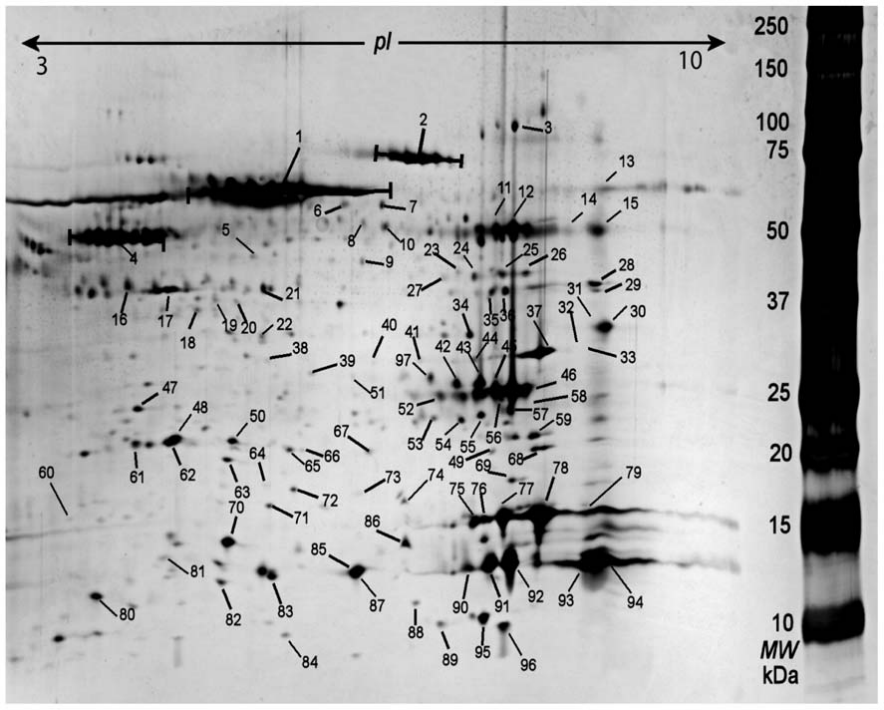
Typical 2-DE pattern from interstitium of trapezius muscle. The numbered protein spots are referred to the identified proteins in Table 1.

**Figure 2:**
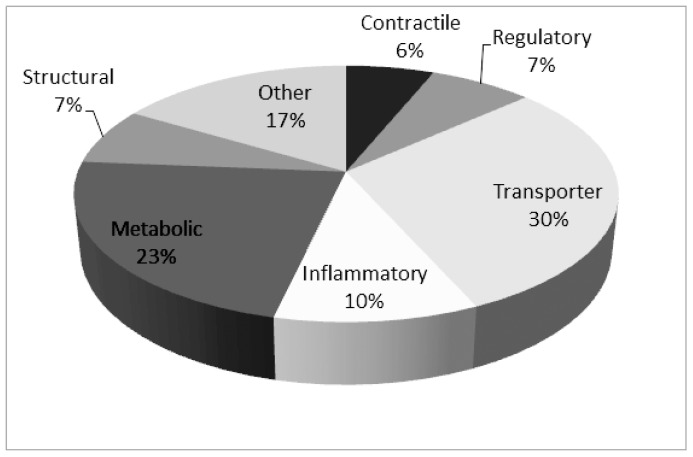
Proportions (in per cent) of different classes for the identified 97 proteins. The categorizations are based on protein function according to Swiss-Prot database.

About 300 protein spots could be detected. 98 protein spots that were of good quality for identification were picked and in-gel digested for identification by nLC- MS/MS and MALDI-TOF mass spectrometer (**fig. 1**, **Table 1**). It was possible to identify 97 of the protein spots. The apparent molecular weight and isoelectric point (p*I*) determined from 2-DE pattern were generally in agreement with the theoretical values with the identified proteins. 50% of the proteins had different identity according to the accession numbers, i.e., many of the identified proteins were expressed as different isoforms. That could be explained by post translational modification and truncation of the proteins. The majority of identified proteins are known muscle proteins pertaining to several functional classes, i.e., metabolic, structural, regulatory, and contractile proteins and proteins that are involved in inflammatory responses (**fig. 2**). Identified proteins known to be involved in nociceptive and pain processes were alpha-1 antitrypsin, creatine kinase, nerve grow factor, carbonic anhydrase, myoglobin, fatty acid binding protein and actin aortic smooth muscle.

**Table 1:** Identified proteins in dialysate samples from microdialysis of the trapezius muscle using a catheter with 100 kDa cut-off.

Spot no.	Protein	Molecular Function	Accession No.	Mw(kDa)/p*I*	Matched peptides	MS Score
***Proteins that are involved in inflammatory processes***						
**4**	Alpha-1-antitrypsin	Serine protease inhibitor	P01009	46.9/5.4	17	600
**5**	Protein S100-A14	Calcium binding protein	Q9HCY8	11.7/5.2	2	21
**49**	Phosphatidylethanolamine-binding protein 1	Protease inhibitor/Serine protease inhibitor	P30086	21.1/7.0	2	89
**64**	Haptoglobin	Serine protease homolog	P00738	45.9/6.1	2	98
**66**	Protein DJ-1	Protease	Q99497	20.0/6.3	1	53
**68**	Phosphatidylethanolamine-binding protein 1	Protease inhibitor	P30086	21.1/7.0	7	288
**82**	Protein S100-A9	Calcium binding protein	P06702	13.3/5.7	4	122
**83**	Protein S100-A9	Ca-binding protein	P06702	13.3/5.7	5	145
**88**	Beta-2-microglobulin	Other	P61769	13.8/6.1	1	50
**97**	Beta-nerve growth factor	Growth factor activity	P01138	27.0/9.8	5	218
***Transporter proteins***						
**1**	Serum albumin	Ca^2+^/Na+/K+/fatty acids/drug binding protein	P02768	71.3/5.9	25	761
**2**	Serotransferrin	Iron binding protein	P02787	79.3/6.8	12	451
**6**	Serum albumin	Ca^2+^/Na+/K+/fatty acids/drug binding protein	P02768	71.3/5.9	7	175
**8**	Serum albumin	Ca^2+^/Na+/K+/fatty acids/drug binding protein	P02768	71.3/5.9	8	213
**9**	Serum albumin	Ca^2+^/Na+/K+/fatty acids/drug binding protein	P02768	71.3/5.9	7	221
**19**	Serum albumin	Ca^2+^/Na+/K+/fatty acids/drug binding protein	P02768	71.3/5.9	3	59
**21**	Serum albumin	Ca^2+^/Na+/K+/fatty acids/drug binding protein	P02768	71.3/5.9	7	153
**22**	Serum albumin	Ca^2+^/Na+/K+/fatty acids/drug binding protein	P02768	71.3/5.9	8	221
**32**	Myoglobin	Muscle protein	P02144	17.2/7.1	5	193
**34**	Vacuolar protein sorting-associated protein 28 homolog	Regulation of vesicular trafficking	Q9UK41	25.6/5.3	5	34
**48**	Retinol-binding protein 4	Other	P02753	23.3/5.8	2	47
**56**	Serum albumin	Ca^2+^/Na+/K+/fatty acids/drug binding protein	P02768	71.3/5.9	6	161
**61**	Apolipoprotein A-1	Lipid metabolism	P02647	30.7/5.6	8	267
**62**	Apolipoprotein A-1	Lipid metabolism	P02647	30.7/5.6	8	331
**70**	Transthyretin	Thyroid hormone-binding	P02766	16.0/5.5	4	186
**75**	Myoglobin	Muscle protein	P02144	17.2/7.1	6	195
**76**	Myoglobin	Muscle protein	P02144	17.2/7.1	4	135
**77**	Myoglobin	Muscle protein	P02144	17.2/7.1	5	277
**78**	Myoglobin	Muscle protein	P02144	17.2/7.1	7	432
**79**	Myoglobin	Muscle protein	P02144	17.2/7.1	4	201
**81**	Syntaxin-8	Transport	Q9UNK0	27.0/4.9	1	31
**85**	Fatty acid-binding protein	Lipid transport	P05413	14.9/6.3	7	227
**87**	Fatty acid-binding protein	Lipid transport	P05413	14.9/6.3	10	312
**89**	Hemoglobin subunit beta	Hypotensive agent	P68871	16.1/6.7	1	62
**90**	Chain B, Deoxy hemoglobin	Hypotensive agnet	gi|27574248	15.9/6.7	4	193
**91**	Hemoglobin subunit beta	Hypotensive agent	P68871	16.1/6.7	7	514
**92**	Hemoglobin subunit beta	Hypotensive agent	P68871	16.1/6.7	12	724
**93**	Hemoglobin subunit alpha	Other	P69905	15.3/8.7	5	803
**94**	Hemoglobin subunit alpha	Other	P69905	15.3/8.7	6	229
***Contractile proteins***						
**10**	Actin, cytoplasmic 1	Other	P60709	42.0/5.3	2	48
**16**	Actin, aortic smooth muscle	Muscle protein	P62736	42.4/5.2	2	83
**17**	Actin, aortic smooth muscle	Muscle protein	P62736	42.4/5.2	9	243
**18**	Myosin light chain 5	Muscle protein	Q02045	19.5/4.9	3	376
**60**	Calponin-3	Actin binding protein	Q15417	36.4/5.7	4	981
**69**	Cofilin-1	Other	P23528	18.7/8.2	1	27
***Regulatory proteins***						
**7**	Glomulin	Muscle cell differentiation	Q92990	68.9/5.2	1	47
**41**	rRNA methyltransferase 1, mitochondrial	Methyltransferase	Q6IN84	38.6/8.2	11	1.06e+6
**47**	Rho GDP-dissociation inhibitor2	GTPase activation	P52566	23.0/5.1	5	166
**50**	Glutathione S-transferase P	Transferase	P09211	23.6/5.4	5	275
**73**	Transforming growth factor beta-1	Growth factor/Mitogen	P01137	44.3/8.8	5	679
**74**	Host cell factor C1 regulator 1	Other	Q9NWW0	15.4/6.8	1	58
**80**	26S protease regulatory subunit 4	ATP binding protein	P62191	49.2/5.9	6	104
***Structural proteins***						
**20**	Keratin, type I cytoskeletal 9	Other	P35527	62.2/5.1	4	195
**30**	Four and a half LIM domains protein 1	Developmental protein	Q13642	38.0/9.2	2	86
**31**	Four and a half LIM domains protein 1	Developmental protein	Q13642	38.0/9.2	1	75
**33**	Four and a half LIM domains protein 1	Developmental protein	Q13642	38.0/9.2	1	68
**37**	Four and a half LIM domains protein 1	Developmental protein	Q13642	38.0/9.2	1	59
**38**	Keratin, type I cytoskeletal 9	Other	P35527	62.2/5.1	12	746
**58**	Four and a half LIM domains protein 1	Developmental protein	Q13642	38.0/9.2	1	48
***Metabolic proteins***						
**3**	Carbonic anhydrase 3	Lyase	P07451	29.8/6.9	5	142
**11**	Carbonic anhydrase 3	Lyase	P07451	29.8/6.9	5	178
**12**	Carbonic anhydrase 3	Lyase	P07451	29.8/6.9	6	210
**13**	Acyl-coenzyme A synthetase ACSM2A	Ligase	Q08AH3	64.8/8.3	1	37
**23**	Beta-enolase	Lyase	P13929	47.2/7.6	4	223
**25**	Beta-enolase	Lyase	P13929	47.2/7.6	4	211
**26**	Beta-enolase	Lyase	P13929	47.2/7.6	5	211
**28**	Phosphoglycerate kinase 1	Transferase/Kinase	P00558	45.0/8.3	3	81
**29**	Fructose-bisphosphate aldolase A	Lyase	P04075	39.8/8.3	7	257
**35**	Creatine kinase M-type	Transferase/Kinase	P06732	43.3/6.8	9	386
**36**	Creatine kinase M-type	Transferase/Kinase	P06732	43.3/6.8	10	476
**42**	Carbonic anhydrase 1	Lyase	P00915	28.9/6.6	6	174
**43**	Carbonic anhydrase 1	Lyase	P00915	28.9/6.6	8	276
**44**	Glycosyltransferase 1 domain-containing protein 1	Glycotransferase	Q96MS3	38.5/6.0	7	421
**45**	Carbonic anhydrase 3	Lyase	P07451	29.8/6.9	6	206
**46**	Carbonic anhydrase 3	Lyase	P07451	29.8/6.9	7	264
**52**	Carbonic anhydrase 3	Lyase	P07451	29.8/6.9	1	52
**53**	Acetylcholine receptor subunit alpha	Ligand-gated ion channel,	P02708	54.5/5.8	1	39
**54**	Triosephosphate isomerase	Isomerase	P60174	31.0/5.6	5	196
**55**	Triosephosphate isomerase	Isomerase	P60174	31.0/5.6	5	302
**57**	Carbonic anhydrase 3	Lyase	P07451	29.8/6.9	1	42
**84**	Acyl-protein thioesterase 2	Hydrolase	O95372	25.1/6.7	1	37
						
***Others***						
**14**	Ig gamma-1 chain C region	Antigen binding	P01857	36.6/8.5	2	75
**15**	Ig gamma-1 chain C region	Antigen binding	P01857	36.6/8.5	5	122
**24**	Centromere protein M	Mitotic progression	Q9NSP4	19.7/6.7	2	12
**27**	E3 ubiquitin-protein ligase RNF149	Ligase	Q8NC42	43.1/6.1	1	40
**39**	E3 ubiquitin-protein ligase RNF149	Ligase	Q8NC42	43.1/6.1	1	36
**40**	WD repeat domain phosphoinositide-interacting protein 3	phosphatidylinositol-3,5-bisphosphate binding	Q5MNZ6	38.1/7.5	1	32
**51**	Carboxypeptidase-like 3	Metalloprotease	O75976	44.7/6.5	1	33
**59**	Flavin reductase (NADPH)	Oxidoreductase	P30043	22.2/7.1	3	133
**63**	Histone H2A.2	DNA-binding	P04908	14.2/11	1	44
**65**	DDB1- and CUL4-associated factor 8	Other	Q5TAQ9	67.5/5.2	1	29
**67**	Apoptosis-associated speck-like protein containing a CARD	Apoptosis	Q9ULZ3	21.7/5.9	1	87
**71**	Superoxide dismutase [Cu-Zn]	Antioxidant	P00441	16.1/5.7	2	88
**72**	Cysteine and glycine-rich protein 2	Zinc ion binding proteins	Q16527	21.0/9.0	8	9981
**86**	Unnamed protein product	Other	gi|29446	16.0/7.1	2	130
**95**	ADP-ribosyl cyclase 1	Hydrolase	P28907-2	13.8/9.0	1	35
**96**	Ubiquitin-60S ribosomal protein L40	Ribonucleoprotein	P62987	15.0/9.9	5	142

The proteins have been classified with respect to class and molecular function according to Swissprot. The accession number, molecular weight (Mw (kDa))/isoelectric point (pI),are according to Swissprot or NCBI database. The spot no are referred to numbered protein spots in figure 1.

### 2-DE protein patterns in TM, CWP and CON

**Figure 3:**
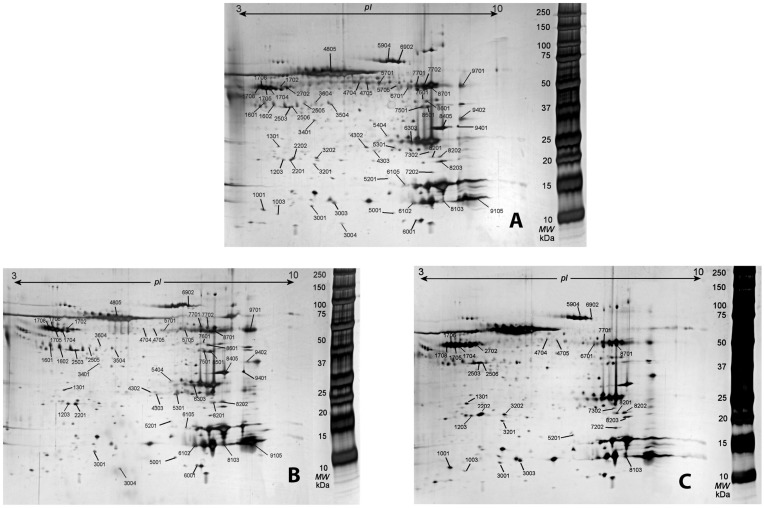
Protein patterns of the muscle dialysate. The 2-DE gels of controls (CON, panel A), chronic trapezius myalgia (TM; panel B), and chronic widespread pain (CWP, panel C). The marked spots are the differentially expressed proteins according to Table 2 and 3.

The protein pattern in the dialysate from CON was almost similar to the pattern in TM and in CWP (**fig. 3**). Higher numbers of protein spots could be detected in TM (262) compared to CWP (196) gel and the healthy control gel (195).

Forty-eight out of 262 proteins in TM (**Table 2**) and 30 out of 196 proteins in CWP (**Table 3**) had concentrations at least two-fold higher or lower than in CON. Seventeen of these proteins showed alterations in the concentrations both in TM and CWP when compared to CON. Twelve out of the 17 proteins were altered in a similar way (at least two-fold higher (↑) or lower (↓)) in both groups of patients compared to CON:

**Table 2:** The optical density for altered proteins (≥twofold up/down regulated) in dialysate from the interstitial fluid of the trapezius in chronic trapezius myalgia (TM) compared to healthy controls (CON).

SSP	Protein	TM	CON
1203	Apolipoprotein AI	1526.9	345.8
1301	Rho GDP-dissociation inhibitor2	604.3	265.7
1601	Actin, aortic smooth muscle	2839.5	1411.2
1602	Actin, aortic smooth muscle	4033.9	1781.2
1702	Alpha-1-antitrypsin	2596.4	882.5
1704	Alpha-1-antitrypsin	10548.6	2477.5
1705	Alpha-1-antitrypsin	1835.1	4911.3
1706	Alpha-1-antitrypsin	8631.6	3950.6
1708	Alpha-1-antitrypsin	5425.6	2585.2
2201	Apolipoprotein A-1	3042.8	891.0
2503	Actin, aortic smooth muscle	873.0	157.4
2505	Serum albumin	211.9	609.9
3001	Protein S100-A9	940.2	463.9
3004	Acyl-protein thioesterase 2	535.8	248.2
3401	Serum albumin	209.3	429.7
3504	Serum albumin	195.9	552.8
3604	Keratin, type I cytoskeletal 9	211.6	807.1
4302	Carbonic anhydrase 3	205.8	791.6
4303	Acetylcholine receptor subunit alpha	681.6	330.2
4704	Serum albumin	201.7	896.0
4705	Serum albumin	171.3	2356.3
4805	Serum albumin	4958.1	11312.5
5001	Beta-2-microglobulin	535.7	152.8
5201	Host cell factor C1 regulator 1	273.0	79.1
5301	Triosephosphate isomerase	5092.9	624.9
5404	Beta-nerve growth factor	232.9	527.3
5701	Actin, cytoplasmic 1	753.7	2737.6
5705	Carbonic anhydrase 3	3655.2	1046.3
6001	ADP-ribosyl cyclase 1	799.9	321.4
6102	Chain B, Deoxy hemoglobin	10021.0	2834.8
6105	Myoglobin	480.4	1075.2
6303	Carbonic anhydrase 1	3526.5	8509.4
6902	Serotransferrin	3102.4	7660.9
7501	Creatine kinase M-type	2284.1	933.3
7601	Beta-enolase	303.4	1074.5
7701	Carbonic anhydrase 3	4035.0	1253.1
7702	Carbonic anhydrase 3	4615.4	10836.0
8103	Hemoglobin subunit beta	13345.5	1213.3
8201	Flavin reductase (NADPH)	1514.9	565.3
8202	Flavin reductase (NADPH)	2456.0	704.0
8405	Four and a half LIM domains protein 1	1138.9	2494.5
8501	Creatine kinase M-type	4416.1	1596.1
8601	Beta-enolase	2580.2	1264.8
8701	Carbonic anhydrase 3	15598.1	3562.7
9105	Hemoglobin subunit alpha	63347.9	29012.7
9401	Four and a half LIM domains protein 1	1552.0	706.8
9402	Four and a half LIM domains protein 1	1256.5	5112.5
9701	Ig gamma-1 chain C region	19206.2	6056.2

The SSP numbers are referred to the spot ide in figure 3A-C. The values are given in part per million (ppm).

**Table 3:** The optical density for altered proteins (≥twofold up/down regulated) in dialysate from the interstitial fluid of the trapezius in chronic widespread myalgia (CWP) compared to healthy controls (CON).

SSP	Protein	CWP	CON
1001	Protein S100-A14	2347.4	849.4
1003	Syntaxin-8	415.9	125.7
1203	Apolipoprotein AI	1200.8	345.8
1301	Rho GDP-dissociation inhibitor2	1280.1	265.7
1704	Alpha-1-antitrypsin	98.5	2477.5
1705	Alpha-1-antitrypsin	17217.1	4911.3
1706	Alpha-1-antitrypsin	1672.9	3950.6
1708	Alpha-1-antitrypsin	7056.7	2585.2
2202	Apolipoprotein A-1	4468.5	1366.7
2503	Actin, aortic smooth muscle	58.3	157.4
2506	Serum albumin	2552.5	1214.7
2702	Alpha-1-antitrypsin	7331.1	2935.8
3001	Protein S100-A9	983.3	463.9
3003	Protein S100-A9	2912.9	1308.2
3201	Histone H2A.2	895.8	274.8
3202	Glutathione S-transferase P	1701.4	646.9
4704	Serum albumin	315.6	896.0
4705	Serum albumin	421.8	2356.3
5201	Host cell factor C1 regulator 1	411.6	79.1
5904	Serotransferrin	19626.1	4499.9
6701	Carbonic anhydrase 3	1182.2	2518.9
6902	Serotransferrin	1508.9	7660.9
7202	Cofilin-1	818.8	380.7
7302	Carbonic anhydrase 3	946.9	213.5
7701	Carbonic anhydrase 3	3331.8	1253.1
8103	Hemoglobin subunit beta	4326.8	1213.3
8201	Flavin reductase (NADPH)	1133.7	565.3
8202	Flavin reductase (NADPH)	2208.9	704.0
8203	Phosphatidylethanolamine-binding protein 1	1035.6	2450.5
8701	Carbonic anhydrase 3	1485.2	3562.7

The SSP numbers are referred to the spot ide in figure 3A-C. The values are given in part per million (ppm).

1. Apolipoprotein AI (SSP 1203; ↑)
2. Rho GDP-dissociation inhibitor2 (SSP 1301; ↑)
3. Alpha-1-antitrypsin (SSP 1708; ↑)
4. Protein S100-A9 (SSP 3001; ↑)
5. Host cell factor C1 regulator 1(SSP 5201; ↑)
6. Carbonic anhydrase 3 (SSP 7701; ↑)
7. Hemoglobin subunit beta (SSP 8103; ↑)
8. Flavin reductase (NADPH) (SSP 8201; ↑)
9. Flavin reductase (NADPH) (SSP 8202; ↑)
10. Serum albumin (SSP 4704; ↓)
11. Serum albumin (SSP 4705; ↓)
12. Serotransferrin (SSP 6902; ↓)

The alterations of the remaining five proteins were different in the two groups (at least two-fold higher or lower) when compared to CON:

1. Alpha-1-antitrypsin (SSP 1704; CWP ↓, TM ↑)
2. Alpha-1-antitrypsin (SSP 1706; CWP ↓, TM ↑)
3. Actin, aortic smooth muscle (SSP 2503; CWP ↓, TM ↑) )
4. Carbonic anhydrase 3 (SSP 8701; CWP ↓, TM ↑))
5. Alpha-1-antitrypsin (SSP 1705;CWP ↑, TM ↓)

## Discussion

We, for the first time to the best of our knowledge, have described the molecular pattern of protein expression in human muscle microdialysate by using two dimensional gel electrophoresis in combination with mass spectrometry. To investigate the possibility that specific proteins in muscle dialysate samples might be markers of the different pain conditions, we analyzed proteins that were changed two folds or more between the three groups. Pronounced alterations in the proteome in the myalgic muscle of the two common chronic pain conditions were found. Hence, major results were:

- A large number of proteins (approx. 300) were found in the interstitium of human muscles using MD in combination with 2-DE analysis.
- The identified proteins (n = 97) were proteins involved in inflammatory processes and metabolic, structural, regulatory, contractile and transporter proteins.
- Considerable proportions of the identified proteins were at least two-fold higher or lower in TM (50%) and CWP (31%) than in CON.
- The two groups of patients (TM and CWP) showed at least two-fold alterations in concentrations of the same proteins (18%) when compared to CON; approx 2/3 of these alterations were in the same direction.

There is a need to understand the activated nociceptive mechanisms at various levels of the pain systems in chronic myalgia. In the present study we have focused upon peripheral alterations and collected dialysate from the interstitium of the trapezius. Earlier MD studies have reported peripheral alterations in concentrations of algesic, metabolic and antinociceptive substances; see [6] for a review. However, these studies have, due to the small volumes of dialysate obtained from the muscle interstitium, only been able to analyze a few substances. The present technique, combining MD, 2-DE analysis and nLC-MS/MS, opens up for an explorative approach not focusing upon predetermined substances, in order to understand the involved mechanisms in the myalgic muscle. Proteins that are involved in pain are important to investigate due to the fact that they can act as signal substances, activate the formation of algesics and control nociceptive processes. The number of studies investigating the human proteome in body fluids and tissues in human *chronic* pain conditions are limited e.g., [17]–[22], [35]–[37]. There are also a few examples of proteomic studies in acute pain e.g., acute coronary syndrome [38], after eccentric exercises [39] and experimental cutaneous injury [40]. In the latter study details about the proteome of healthy human dermal dialysate in response to acute injury were reported. The authors suggested that the combination of microdialysis and proteomics has the potential to identify relevant, novel markers of injury and inflammation. The present results based upon two different groups of patients with chronic pain extend their suggestion to also include peripheral nociceptive processes and their consequences.

A number of previous proteomic studies of human skeletal muscle biopsies have reported identification of more proteins compared to this study [41], [42], but there are limitations to the analysis of the entire muscle proteome [39]. The high abundant proteins, i.e. contractile, structural and metabolic proteins, impair the detection of low abundant proteins such as inflammatory proteins. Using MD approximately 300 proteins were found in the dialysate of the muscle interstitium of the trapezius muscle. 10% of the identified proteins in this study are proteins that are involved in inflammatory processes (**fig. 2** and **table 1**). The total protein concentrations and the number of protein spots were higher in TM compared to CWP and CON. Several of the identified proteins presented in **table 1** have previously been reported to be involved in nociceptive and pain processes (inflammatory and non-inflammatory pain) such as: creatine kinase [43], nerve growth factor [44], carbonic anhydrase [45], myoglobin [46], fatty acid binding protein [47] and actin aortic smooth muscle [48]. Hence, the presented methodology has a potential of identifying mechanisms involved in nociceptive processes in chronic muscle pain. Moreover, 30 proteins in CWP (**table 3**) and 48 proteins in TM (**table 2**) out of the 196 and 262 protein spots respectively, had concentrations at least twofold higher or lower than in CON. In other words a relatively prominent part of the identified proteins showed marked alterations in TM and CWP when compared to CON. This finding underscores that chronic myalgia, both as a part of a regional pain condition (TM) or a widespread pain condition (CWP), is associated with pronounced alterations in the proteome of the trapezius muscle. Some of these alterations are linked to chronic nociceptive processes while others reasonably are linked to the consequences of being in persistent pain e.g., deconditioning. Another alternative, very briefly reviewed by Gill et al, is that the insertion of microdialysis probes causes long-lasting tissue alterations [40]. In the present study the insertion procedure of the microdialysis probe was standardized and conducted in the same way in all three groups. Thus the differences reported in **tables 2** and **3** are reasonably mainly due to the two former mechanisms. By combining **tables 2** and **3** it was obvious that 17 of the identified proteins showed alterations in the concentrations both in TM and CWP when compared to CON; several of these were categorized as proteins involved in inflammatory processes ( table 1) In twelve out of the 17 proteins the direction of the alterations were similar in the two groups of patients with chronic myalgia and in the remaining five proteins the two groups had different signs of the alterations when compared to the gel of CON. These results indicate both common and different mechanisms in the two pain conditions.

Several of the altered spots were identified as the same protein, suggesting the presence of isoforms. This result is not surprising since many proteins are undergoing post translational modifications particularly involving glycosylation. Alpha-1 antitrypsin (A1AT) was one of the altered proteins that were detected as several different isoforms (SSP; 1704, 1705, 1706 and 1708) depending on the glycosylation [49]. The directions of alterations were different for the different isoforms. A1AT is an acute-phase serine proteinase inhibitor with a broad anti-inflammatory spectrum and it has been suggested that it controls the inflammatory component of musculoskeletal connective tissue associated with fibromyalgia [50].The results from two independent series of genotyping 3127 subjects for A1AT supported the hypothesis that the 13% of persons carrying A1AT polymorphisms may represent almost 40% of all cases of fibromyalgia [51].

Elevated levels of S100 protein family (S100-A8, S100-A9 and S100-A12) have been demonstrated in several inflammatory conditions, both locally at sites of inflammation and in the circulation [52]. These proteins are termed calgranulins, reflecting calcium-binding properties and high expression in granulocytes. A normalization of S100A8/9 levels in RA patients who achieved remission shortly after the initiation of conventional treatment have been demonstrated [53]; the decrease in S100A8/9 rather than CRP levels were associated with improvements in the total number of swollen joints over time.

N-acylethanolamines (NAEs) belong to a family of lipids that are able to activate different receptor systems, including nuclear PPAR-alpha receptors. Fatty acid binding proteins (FABPs) are essential for efficient intranuclear NAE trafficking and activation of PPAR-alpha receptors. FABPs also mediate endocannabinoid hydrolysis by FAAH [54]. FABP (spot no. 85 and 87) and phosphatidylethanolamine-binding protein (spot no 49 and 68) could be identified in the muscle dialysate expressed as two isoforms with different isoelectric points. We have previously reported elevated levels of NAEs in the trapezius dialysate in women with chronic trapezius myalgia [55]. In future studies it would be of high importance to investigate the expression levels of FABP and phosphatedylethanolamine-binding protein in dialysate from TM and CWP. Increased level of phosphatidylethanolamine-binding protein has been reported in the *vastus lateralis* muscle after 55 days of immobilization [56]. FABP has been suggested as a marker for the skeletal muscle injury induced by eccentric exercise [47].

We have also identified a number of proteins that are involved in the oxidative processes in muscle; carbonic anhydrase III, Superoxide dismutase [Cu-Zn], glutathione S-transferase P and flavin reductase (NADPH). Oxidative stress and contractile dysfunction of skeletal muscle induced by reactive oxygen species (ROS) result an imbalance of cellular redox potential and has profound effects on protein carbonylation [57] that leads to modifications in amino acid side chains that result in altered structure and/or functions of the proteins. Changes of the oxidation level of carbonic anhydrase III with muscle unloading have been reported [45]. NADPH is a main source of ROS generation inside skeletal muscle fibers and it has been suggested that the NADPH derived ROS play important physiological roles in regulating skeletal muscle signaling [58]. Superoxide dismutase [Cu-Zn] is an antioxidant protein present in the sarcoplasm of the skeletal muscle fibers and neutralizes ROS production to protect the skeletal muscle against oxidative stress. It has been reported that deficiency in superoxide dismutase [Cu-Zn] results in skeletal muscle weakness and functional innervation [59]. Glutathione S-transferase function also as an antioxidant agent in skeletal muscle, significantly higher level of the antioxidant has been observed in *vastus lateralis* biopsies from patient with chronic fatigue syndrome [60].

Further protein that was identified in muscle microdialysis and that was differentially expressed in TM and CWP compared to CON was Apolipoprotein AI (SSP no 1203, figure 3). Apolipoprotein AI is involved in lipid transport between cells during regeneration and degeneration of neurons [61] and has been suggested as a biomarker for neuropathic pain [11]. Proteomic study of skeletal muscle lipid droplets has shown that Apolipoprotein AI is expressed endogenously by skeletal muscle cells [62]. Apolipoprotein AI is also associated with acute-phase response and promotes anti-inflammatory and antioxidant effects [63], [64]. It has been identified as a biomarker of acute painful episodes [64].

Four and a half LIM domains protein 1 (FHL-1) is highly expressed in skeletal muscle and has been suggested to be a regulator of myogenesis and muscle growth [65], [66]. We identified this protein as intense spots (spot no. 30, 31, 33, 37) on the 2-DE proteome map of interstitium of trapezius muscle that can confirm that the identified proteins are typical muscle protein as FHL-1 is key skeletal muscle protein. FHL-1 has been identified as a regulator of skeletal muscle mass and suggested as a novel therapeutic target in muscle myopathy and atrophy [67] and it has also been identified in oxidative stress conditions in skeletal muscle [45].

There are limitations with the present technique of MD and 2-DE. The most important is the use of pooled samples from several participants to get the acquired amount of protein needed for 2-DE. Another limitation is that MD is an invasive technique although, according to our experience, most subjects perceive it as minimally invasive. In future studies it is important to optimize the relative recovery, the flow rate of the perfusate/dialysate and the composition of the perfusate in order to achieve a more complete picture of the proteome of the muscle interstitium. By extending the microdialysis time to collect more dialysate and proteins, this would be enough to make a separate analysis per participant making it possible to quantify individual differences. Another limitation is that food intake before the microdialysis not was controlled for. In future studies this can be achieved by a standardized overnight fasting and standardized breakfast before the experiment.

With respect to future perspectives proteins identified from this microdialysis study together with proteomic results from muscle biopsy could be a useful approach to find out the biological meaning of the identified proteins as valuable markers of different state of musculoskeletal pain. Further study on the protein alteration over time during the microdialysis experiment (before and after low force repetitive work) and of the recovery of the altered proteins would be the next intressting step.

In conclusion, 97 protein spots were identified from the interstitial fluid of the trapezius muscle and several of these are known to be involved in nociceptive processes. Forty-eight proteins in TM and 30 proteins in CWP had concentrations at least two-fold higher or lower than in CON. Investigating muscle dialysate using the applied techniques in combination (in vivo MD, 2-DE and mass spectrometry) open up for a) the possibility of investigating protein changes associated with different disease processes of musculoskeletal pain e.g., in chronic trapezius myalgia and in chronic widespread myalgia, b) a better understanding of the pathophysiological mechanisms of chronic muscle pain and c) to identify clinically applicably biomarkers.
